# Untangling dopamine-adenosine receptor-receptor assembly in experimental parkinsonism in rats

**DOI:** 10.1242/dmm.018143

**Published:** 2014-11-14

**Authors:** Víctor Fernández-Dueñas, Jaume J. Taura, Martin Cottet, Maricel Gómez-Soler, Marc López-Cano, Catherine Ledent, Masahiko Watanabe, Eric Trinquet, Jean-Philippe Pin, Rafael Luján, Thierry Durroux, Francisco Ciruela

**Affiliations:** 1 Unitat de Farmacologia, Departament Patologia i Terapèutica Experimental, Facultat de Medicina, IDIBELL-Universitat de Barcelona, L’Hospitalet de Llobregat, 08907 Barcelona, Spain.; 2 Institut de Génomique Fonctionnelle, CNRS, UMR5203, Montpellier, France.; 3 INSERM, U.661, Montpellier and Université Montpellier 1,2, Montpellier, F-34094, France.; 4 IRIBHM, Université Libre de Bruxelles, B1070 Brussels, Belgium.; 5 Department of Anatomy, Hokkaido University School of Medicine, Sapporo 060-8638, Japan.; 6 Cisbio Bioassays, 30200 Codolet, France.; 7 Instituto de Investigación en Discapacidades Neurológicas (IDINE), Dept Ciencias Médicas, Facultad de Medicina, Universidad Castilla-La Mancha, 02006 Albacete, Spain.

**Keywords:** Immunoelectron microscopy, Oligomerization, Parkinson’s disease, Proximity ligation assay, TR-FRET

## Abstract

Parkinson’s disease (PD) is a dopaminergic-related pathology in which functioning of the basal ganglia is altered. It has been postulated that a direct receptor-receptor interaction – i.e. of dopamine D_2_ receptor (D_2_R) with adenosine A_2A_ receptor (A_2A_R) (forming D_2_R-A_2A_R oligomers) – finely regulates this brain area. Accordingly, elucidating whether the pathology prompts changes to these complexes could provide valuable information for the design of new PD therapies. Here, we first resolved a long-standing question concerning whether D_2_R-A_2A_R assembly occurs in native tissue: by means of different complementary experimental approaches (i.e. immunoelectron microscopy, proximity ligation assay and TR-FRET), we unambiguously identified native D_2_R-A_2A_R oligomers in rat striatum. Subsequently, we determined that, under pathological conditions (i.e. in a rat PD model), D_2_R-A_2A_R interaction was impaired. Collectively, these results provide definitive evidence for alteration of native D_2_R-A_2A_R oligomers in experimental parkinsonism, thus conferring the rationale for appropriate oligomer-based PD treatments.

## INTRODUCTION

The striatum, the key brain area in the neurobiology of Parkinson’s disease (PD), receives the densest dopamine innervation and thus holds the highest concentration of dopamine in the central nervous system (Gerfen, 2004). It contains two main types of neurons, the GABAergic dynorphinergic neurons, primarily expressing dopamine D_1_ receptors (D_1_Rs), and the GABAergic enkephalinergic neurons, which predominantly express dopamine D_2_ receptors (D_2_Rs) (Gerfen et al., 1990). The control of this brain area is not only limited to dopamine receptors, but also to other G protein-coupled receptors (GPCRs), such as glutamate and adenosine receptors, which impinge into the dopaminergic system by means of functional and molecular interactions, a fact that might allow a fine-tuning modulation of the basal ganglia (Ferré et al., 2004). Conversely, the development of a dopaminergic-related pathology (i.e. PD) prompts changes to these structures, thus impeding proper striatal functioning (Fuxe et al., 2010).

It has been postulated that a direct receptor-receptor interaction occurs between D_2_Rs and adenosine A_2A_ receptors (A_2A_Rs) within GABAergic enkephalinergic neurons (Ferré et al., 1997; Fuxe et al., 1998; Torvinen et al., 2002). However, although direct molecular evidences of D_2_R-A_2A_R oligomerization have been extensively shown in heterologous expression systems (Cabello et al., 2009; Canals et al., 2003; Ciruela et al., 2004; Kamiya et al., 2003), to our knowledge it has still not been definitely proved, despite certain data pointing to it, in native tissue (i.e. striatum). Thus, by co-immunoprecipitation experiments it was possible to observe D_2_R-A_2A_R association in rat striatum homogenates (Cabello et al., 2009). In addition, the precise simultaneous distribution of D_2_Rs and A_2A_Rs in striatal neurons was shown by means of immunoelectron microscopy (Cabello et al., 2009). Furthermore, the feasibility of D_2_R-A_2A_R oligomerization in striatum from mice and monkeys was recently shown by means of the proximity ligation assay (PLA) (Bonaventura et al., 2014; Trifilieff et al., 2011), and from rats by radioligand binding experiments (Pinna et al., 2014). Importantly, these last studies proposed that D_2_R-A_2A_R oligomer formation would be disrupted in parkinsonian L-DOPA-treated animals (Bonaventura et al., 2014; Pinna et al., 2014).

In order to unequivocally demonstrate GPCR oligomerization, the use of fluorescence resonance energy transfer (FRET)-based approaches is now widely accepted (Ciruela et al., 2010). Interestingly, a time-resolved FRET (TR-FRET)-based approach was recently developed to study receptor-receptor interactions under physiological conditions (for a review, see Cottet et al., 2013). In brief, this approach consists of the non-covalent labelling of receptors with selective ligands and/or antibodies bearing compatible fluorophores to engage in a TR-FRET process. It was then possible to provide evidence of oxytocin receptor dimers in rat mammary gland, and later on of D_2_R-ghrelin heteromers in mouse hypothalamus (Albizu et al., 2010; Kern et al., 2012). Here, we aimed to reveal in a similar way native D_2_R-A_2A_R oligomerization. Accordingly, as well as taking advantage of distinct and complementary techniques (i.e. immunoelectron microscopy and PLA), we developed fluorescent ligands for D_2_Rs and A_2A_Rs, and the direct receptor-receptor interaction was evidenced by TR-FRET measurements in striatal preparations. Furthermore, we also investigated whether pathology (i.e. the 6-OHDA rat model of PD) prompted changes in oligomer formation, which would correlate with some of the observed PD pathophysiological features, and thus provide a rationale for appropriate D_2_R-A_2A_R oligomer-based pharmacotherapy.

**TRANSLATIONAL IMPACT**

**Clinical issue**

Parkinson’s disease (PD) is the second most common neurodegenerative disorder. Although its treatment relies mostly on dopamine-like drugs, the introduction of non-dopaminergic strategies as part of the PD therapeutic armamentarium is being increasingly recognized. Indeed, adenosine A_2A_ receptor (A_2A_R) antagonists belong to these kinds of drugs that are currently proposed to target difficult-to-treat PD symptoms. However, the molecular rationale for the pharmacological use of such compounds is still poorly understood.

**Results**

In this study, a multi-methodological approach was designed to demonstrate the direct interaction of dopamine D_2_ receptor (D_2_R) with A_2A_R in a brain region that is important in PD, the striatum. Control and unilateral 6-OHDA-lesioned rats (a widely used animal model of PD) were used. Thus, D_2_R-A_2A_R oligomerization status was assessed in healthy and diseased striatal membranes, and a significant reduction of the D_2_R-A_2A_R oligomer content was observed in the striatum of 6-OHDA-lesioned rats.

**Implications and future directions**

The D_2_R-A_2A_R oligomer disruption reported in this study could represent a striatal hallmark of PD, which indeed could denote a neuroadaptive response to the well-known loss of dopaminergic neurotransmission in PD. Hence, the obtained results support a multimodal dopamine-adenosine approach for PD management: selectively targeting the D_2_R-A_2A_R oligomerization status via combined pharmacotherapeutic strategies could restore the unbalanced D_2_R-A_2A_R oligomer function associated with PD.

## RESULTS

**Fig. 1.**
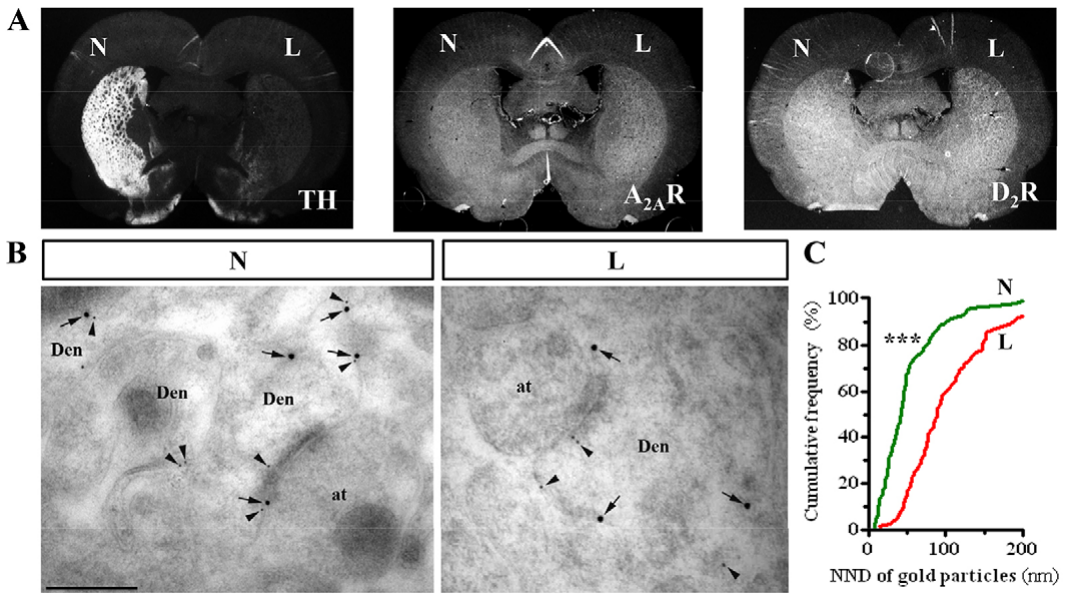
**D_2_R-A_2_R co-clustering in the striatum of normal and 6-OHDA-lesioned rats.** (A) Photomicrographs showing, by means of TH staining, (left panel) the loss of dopaminergic innervation in the lesioned dorsomedial striatum (L) compared with the non-lesioned (N) striatum, and also the expression of A_2A_R (middle) and D_2_R (right) in 6-OHDA-lesioned rat brain coronal slices. (B) Electron micrographs from normal (N) and lesioned (L) hemispheres from 6-OHDA-lesioned rats showing immunoreactivity for A_2A_R and D_2_R in striatum as revealed using a double-labelling post-embedding immunogold technique. Immunoparticles A_2A_R (10-nm size, arrowheads) and D_2_R (15-nm size, arrows) were detected along the extrasynaptic and perisynaptic plasma membrane of the same dendritic shafts (Den) establishing excitatory synaptic contact with axon terminals (at). Scale bar: 0.2 μm. (C) Quantitative analysis of the spatial distance between A_2A_R and D_2_R, which indicated significant differences between normal and 6-OHDA-lesioned rats (***P<0.001). NND, nearest neighbour distance.

The aim of the present work was to: (1) unambiguously demonstrate the existence of D_2_R-A_2A_R oligomers in the rat striatum, and (2) assess possible oligomer alterations under pathological conditions. To this end we used the unilateral 6-OHDA-lesioned rat, a classic and widespread toxin-based animal model of experimental parkinsonism (Schwarting and Huston, 1996). First, we analyzed the extent of the 6-OHDA lesion in our behaviourally selected hemiparkinsonian rats (see Materials and Methods) (Hodgson et al., 2009) by means of immunohistochemistry detection of tyrosine hydroxylase (TH). As expected, a significant reduction of TH expression in the lesioned hemisphere was observed in these animals, thus suggesting a loss of dopaminergic neurons (Fig. 1A). Similarly, A_2A_R and D_2_R expression was also assessed and a high and consistent striatal expression observed, as previously demonstrated (Levey et al., 1993; Rosin et al., 2003). It is important to mention here that we validated the specificity of the D_2_R and A_2A_R antibodies used. Thus, A_2A_R and D_2_R immunoreactivity was concentrated in the striatum, and no staining was observed either in adjacent cortical areas or in A_2A_R knockout (A_2A_R-KO) mouse tissue (supplementary material Fig. S1). Next, we detected striatal D_2_R and A_2A_R at the subcellular level using double-labelling immunogold electron microscopy in 6-OHDA-lesioned rats. Thus, immunoparticles for D_2_R and A_2A_R showed a high degree of co-distribution in dendritic spines of asymmetric synapses (i.e. glutamatergic terminals) in normal brain from 6-OHDA-lesioned rats (Fig. 1B, left), as previously described in human embryonic kidney (HEK-239) cells (Cabello et al., 2009). Interestingly, when the 6-OHDA-lesioned hemisphere was assessed, a reduction in the co-distribution and proximity of immunoparticles for D_2_R and A_2A_R was observed (Fig. 1B, right). Thus, when D_2_R-A_2A_R co-clustering was quantified in both hemispheres (normal and lesioned) the distances between D_2_R and A_2A_R were significantly increased in the 6-OHDA-lesioned hemisphere (Fig. 1C). Overall, these results suggested that the distribution of D_2_R-A_2A_R oligomers in the rat striatum might be altered under pathological conditions.

**Fig. 2.**
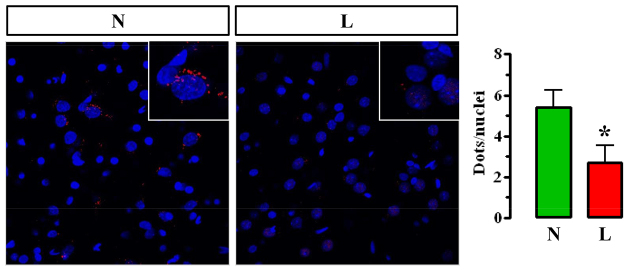
**Detection of D_2_R and A_2_R proximity in normal and 6-OHDA-lesioned rat striatal sections.** Photomicrographs of dual recognition of D_2_R and A_2A_R with proximity ligation assay (PLA) from normal (N) and lesioned (L) hemispheres from 6-OHDA-lesioned rats. Quantification of PLA signals for D_2_R and A_2A_R proximity confirmed the significant difference of PLA signal density between normal and 6-OHDA-lesioned rats (*P<0.05). Values in the graph correspond to the mean ± s.e.m. (dots/nuclei) of at least six animals for each condition.

Next, we aimed to corroborate the former results by detecting D_2_R-A_2A_R complexes using the PLA. We first validated the specificity of this technique by comparing the obtained PLA signal (see Materials and Methods) in wild-type and A_2A_R-KO mice, in which it was not present (supplementary material Fig. S2). Similarly, a PLA signal was virtually absent in cortex from wild-type mice, a result that was also obtained when evaluating the PLA signal in rats, in which fluorescence was dramatically enhanced in striatal slices (supplementary material Fig. S2). Therefore, we performed the PLA in 6-OHDA-lesioned rats. Interestingly, as shown in the immunogold electron microscopy experiments, the PLA signal in the 6-OHDA-lesioned striatal hemisphere was reduced when compared to the non-lesioned striatal hemisphere (Fig. 2). Overall, these results clearly supported the hypothesis that a receptor-receptor interaction between D_2_R and A_2A_R occurs in the striatum and that this interaction might be significantly reduced under pathological conditions.

In order to further demonstrate the existence of native D_2_R-A_2A_R oligomers, we next performed TR-FRET experiments with fluorescent ligands. To this end, we used the D_2_R antagonist N-(p-aminophenethyl)spiperone (NAPS) derived with Lumi4-Tb (NAPS-Lumi4-Tb) as donor, which has been successfully used to detect D_2_R homodimers (Albizu et al., 2010), in combination with the fluorescent A_2A_R antagonist (SCH-red) derived from A_2A_R antagonist SCH442-416 with dy647. Importantly, we first validated this approach on receptors expressed in HEK-293 cells. Using the tag-lite saturation and competition binding assays (supplementary material Fig. S3A) (Zwier et al., 2010), we estimated SCH-red affinity for A_2A_R at 4.8±1.5 nM (supplementary material Fig. S3B), confirming its high affinity for this receptor. The inhibition constant of unlabelled ligand SCH442-416 was 2.9±0.4 nM (supplementary material Fig. S3C), which is in accordance with previously reported data (Müller and Jacobson, 2011). D_2_R- and A_2A_R-transfected cells were then incubated in the presence of the fluorescent ligands (supplementary material Fig. S3D). Thus, we set the NAPS-Lumi4-Tb concentration at 1 nM, as previously described (Albizu et al., 2010), and chose the 10 nM concentration of SCH-red [about 2× the dissociation constant (*K*_d_)] for further TR-FRET experiments. Of note, the challenge was to get the highest occupation of receptor binding sites with ligands but the lowest non-specific TR-FRET. Incubation of cells expressing D_2_R and A_2A_R with both fluorescent ligands led to a significant TR-FRET signal (supplementary material Fig. S3E). The specificity of the TR-FRET signal was based on various data: a signal was neither observed in D_2_R-expressing cells nor in the presence of an excess of unlabelled ligand (SCH442-416 1 μM) (supplementary material Fig. S3E); additionally, TR-FRET signal was saturable when cells were incubated in the presence of a constant concentration of NAPS-Tb and increasing concentrations of SCH-red. Interestingly, the affinity of the SCH-red compound for A_2A_R in D_2_R- and A_2A_R-expressing cells (forming D_2_R-A_2A_R oligomer) was pretty much the same (*K*_d_=4.1±1.2 nM; supplementary material Fig. S3F) as the one found for the A_2A_R expressed alone (see above), thus suggesting that the oligomer formation did not alter the pharmacodynamic properties of A_2A_R.

**Fig. 3.**
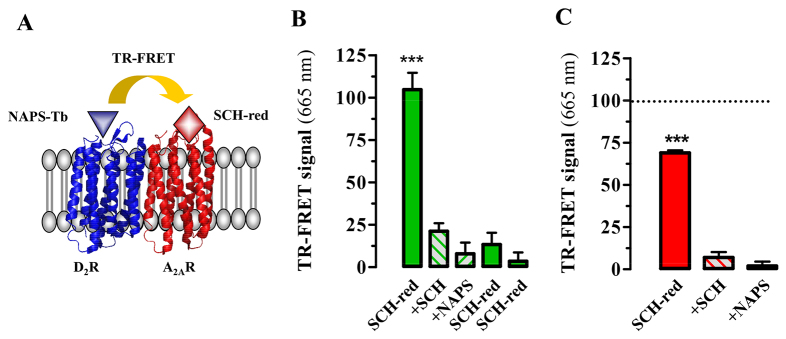
**Evidencing D_2_R-A_2A_R oligomer existence and disruption in rat striatal membranes of normal and 6-OHDA-lesioned rats.** (A) Diagram illustrating the principle of TR-FRET between the fluorescent ligands bound to D_2_R and A_2A_R. (B) Membrane preparations from rat striatum were labelled with NAPS-Lumi4-Tb (1 nM) plus SCH-red (10 nM), which resulted in a TR-FRET signal (1st column) higher than that obtained when labelling membranes with NAPS-Lumi4-Tb (1 nM) plus SCH-red (10 nM) and SCH (1 μM; 2nd column) or plus SCH-red (10 nM) and NAPS (1 μM; 3rd column) (***P<0.001). Membranes from the cortex of the same rats (4th column) and also striatal membranes from KO-A_2A_R mice (5th column) were used as negative controls of the TR-FRET signal, because they were incubated with the fluorescent ligands at the same concentration as in the striatum. (C) Striatal membrane preparations from 6-OHDA-lesioned rats were labelled with NAPS-Lumi4-Tb (1 nM) plus SCH-red (10 nM), and the signal (1st column) was lower when labelling with NAPS-Lumi4-Tb (1 nM) plus SCH-red (10 nM) and SCH (1 μM; 2nd column) or plus SCH-red (10 nM) and NAPS (1 μM; 3rd column) (*P*<0.001). Values correspond to the mean ± s.e.m. (AU, arbitrary units) of 4–6 animals for each condition.

Once we had validated the fluorescent-ligand-based TR-FRET strategy, we attempted to detect the native D_2_R-A_2A_R oligomer in the rat striatum. Accordingly, we obtained rat striatal membranes and processed them for oligomer TR-FRET detection using NAPS-Lumi4-Tb (1 nM) and SCH-red (10 nM) as described previously (Fig. 3A). Owing to low receptor expression levels compared with transient transfected cells, we optimized experimental conditions by performing experiments on fresh membrane preparations purified by means of sucrose gradients. Upon these experimental conditions, incubation of striatal membranes with NAPS-Lumi4-Tb (1 nM) and SCH-red (10 nM) led to a TR-FRET signal (Fig. 3B). The specificity of the signal was supported by two findings. First, an excess of unlabelled antagonists (either NAPS or SCH442-416) blocked the TR-FRET signal. Second, TR-FRET was neither observed in striatal membranes of A_2A_R-KO mice nor in rat cortex membrane preparations, where the expression of D_2_Rs and A_2A_Rs is very low compared with in the striatum (Fig. 3B). Overall, by means of the strategy based on TR-FRET between fluorescent ligands, we were able to provide clear-cut evidence of the existence of D_2_R-A_2A_R oligomers in the rat striatum.

Finally, we aimed to ascertain the previous findings indicating a decrease of D_2_R-A_2A_R oligomer formation under pathological conditions. Thus, striatal membranes from 6-OHDA-lesioned rats were obtained and challenged with the fluorescent ligands. A specific TR-FRET signal was measured after incubating membranes in the presence of NAPS-Lumi4-Tb (1 nM) and of SCH-red (10 nM) (Fig. 3C), as previously described. Interestingly, a close view of the specific TR-FRET signal obtained in the striatal membranes corresponding to the lesioned hemisphere (6-OHDA) showed a significant reduction in the specific TR-FRET signal when compared with both the non-lesioned hemisphere (−37±6%; *P*<0.05) and the striatum from normal rats (−34±5%; *P*<0.05). Overall, these results confirmed a decreased D_2_R-A_2A_R oligomer formation in the 6-OHDA-lesioned striatal area, thus suggesting that impairment in the D_2_R-A_2A_R oligomer interplay could be a parkinsonian hallmark.

## DISCUSSION

Non-dopaminergic drugs (i.e. A_2A_R antagonists) have been recently introduced in the management of PD (for a review, see Vallano et al., 2011). The expected antiparkinsonian effects of these therapies could rely in part on a direct D_2_R-A_2A_R interaction in the striatum. Hence, demonstrating the existence of this receptor-receptor interaction in this brain area and possible changes associated with the neuropathology might be relevant for the effective design of D_2_R-A_2A_R oligomer-based antiparkinsonian drugs. Indeed, this paradigm has been a matter of controversy for a long time, because D_2_R-A_2A_R assembly in the native context had not been demonstrated. Accordingly, in the present work we first aimed to unequivocally reveal D_2_R-A_2A_R complexes in the rat striatum. To this end, we engaged a multimodal approach, in which distinct and complementary tools were used. Of note, the results obtained by means of immunoelectron microscopy and the PLA were consistent with those previously published (Cabello et al., 2009; Trifilieff et al., 2011). However, these experiments do not permit the undeniable proof of D_2_R-A_2A_R oligomerization; thus, we developed a TR-FRET-based strategy, because only this kind of tool permits the demonstration of direct receptor-receptor interactions at the cell surface when expressed at a physiological level (Albizu et al., 2010; Kern et al., 2012; Maurel et al., 2008). By using indirect receptor labelling with fluorescent ligands, Albizu et al. previously demonstrated oxytocin receptor dimerization in the rat mammary gland (Albizu et al., 2010). Here, despite D_2_R and A_2A_R expression in striatum being much lower than that observed for oxytocin receptors in mammary gland, we were able to find specific D_2_R-A_2A_R oligomerization in striatum, not only proving that the strategy remains efficient even for low receptor expression but also definitely substantiating this long-standing unresolved phenomenon.

Once the existence of a striatal D_2_R-A_2A_R complex was finally demonstrated, we attempted to determine whether the D_2_R-A_2A_R oligomer was altered under pathological conditions. Thus, we generated hemiparkinsonian rats by unilateral injection of 6-OHDA in the striatum and monitored the amount of striatal D_2_R-A_2A_R oligomers. Interestingly, this classic and widespread toxin-based animal model of PD has been successfully used in preclinical studies of antiparkinsonian drugs, including A_2A_R antagonists (Hodgson et al., 2009; Pinna, 2009). Therefore, we assayed the three distinct approaches in striatal preparations of 6-OHDA-lesioned rats, in which it was possible to observe a minor D_2_R-A_2A_R oligomer formation when compared with that observed in normal rats. These results contrast to that reported recently in a primate model of PD (Bonaventura et al., 2014). Thus, in MPTP-lesioned monkeys, a similar PLA approach showed that only L-DOPA-treated animals had a modest reduction in the PLA signal (2.3±0.3 vs 1.8±0.2 dots/nuclei comparing control vs L-DOPA-treated MPTP-lesioned monkeys); no reduction (2.5±0.3 dots/nuclei) was observed in non-treated parkinsonian animals (Bonaventura et al., 2014). These differences could be explained by several reasons: for instance, the animal model (monkey vs rat), the lesion (MPTP vs 6-OHDA) or the antibodies used. Indeed, the PLA signal has been shown to be extremely dependent on the sensitivity and/or specificity of the antibodies used (Trifilieff et al., 2011). In our hands, the PLA signal was consistent and in the same range (5.4±0.8 vs 2.6±0.8 dots/nuclei comparing control vs 6-OHDA-lesioned rats) to that described previously for the same pair of receptors (Trifilieff et al., 2011). Furthermore, our negative controls (A_2A_R-KO and cortex) provided 0.5–1.5 dots/nuclei, in agreement with that described previously in rat striatum (Trifilieff et al., 2011). Overall, our PLA clearly corroborated immunoelectron microscopy and TR-FRET results, thus undoubtedly demonstrating that D_2_R-A_2A_R oligomer formation was reduced in experimental parkinsonism.

Overall, our data suggest the existence of molecular mechanisms in 6-OHDA-lesioned rats that might regulate D_2_R-A_2A_R interactions. It could then be hypothesized that, in pathological conditions, in which impairment of the D_2_R-A_2A_R oligomer would occur (i.e. PD), the well-known D_2_R-A_2A_R transinhibition (for a review, see Ferré et al., 1997; Fuxe et al., 2010) would be reduced. In this pathological context, increased functionality of A_2A_R would be expected, as has been proposed in PD (Ramlackhansingh et al., 2011; Varani et al., 2010), thus supporting the therapeutic use of A_2A_R antagonists in PD management (Xu et al., 2005). In line with this, it was shown that, in the dopamine denervated striatum, an increased A_2A_R-mediated signalling occurs (Tanganelli et al., 2004). These results were explained by an enhancement of the inhibitory D_2_R-A_2A_R interaction; however, they could alternatively support the loss of oligomerization leading to increased functionality of A_2A_R. In conclusion, it seems likely that the design of new therapeutic strategies could be potentially based on selectively targeting the D_2_R-A_2A_R oligomerization status and thus finely controlling the function of such receptors.

## MATERIALS AND METHODS

### Reagents

NAPS and SCH442-416 were purchased from Tocris Bioscience (Ellisville, MI, USA). Lipofectamine 2000 was from Invitrogen (Carlsbad, CA, USA). SNAP-Lumi4-Tb (GK), NAPS-Lumi4-Tb and SCH-red were from Cisbio Bioassays (Bagnols-sur-Cèze, France). The primary antibodies used were: rabbit anti-TH polyclonal antibody (Millipore, Temecula, CA, USA), goat anti-A_2A_R and rabbit anti-D_2_R polyclonal antibodies (Frontier Institute Co. Ltd, Shinko-nishi, Ishikari, Hokkaido, Japan).

### Plasmids and transfection

The pRK5 plasmids encoding wild-type D_2_R and A_2A_R subunits tagged with Halo (h-D_2_R; Cisbio Bioassays) or SNAP (ST-A_2A_R; Fernández-Dueñas et al., 2012) proteins were used. Human embryonic kidney (HEK)-293 cells were cultured in DMEM (Invitrogen) supplemented with 10% fetal calf serum (Lonza, Basel, Switzerland) and nonessential amino acids, penicillin and streptomycin (Invitrogen). Cells were transfected by the reverse Lipofectamine 2000 protocol as described by the manufacturer (Invitrogen).

### Animals

Sprague-Dawley rats (Charles River Laboratories, L’Arbresle, France) weighing 240–250 g, and CD-1 mice (Charles River Laboratories) and A_2A_R-KO mice (Ledent et al., 1997) weighing 20–25 g were used. They were housed in standard cages with *ad libitum* access to food and water, and maintained under controlled standard conditions (12 hour dark/light cycle starting at 7:30 am, 22°C temperature and 66% humidity). The University of Barcelona Committee on Animal Use and Care approved the protocol, and the animals were housed and tested in compliance with the guidelines described in the Guide for the Care and Use of Laboratory Animals (Clark et al., 1997) and following the European Community law 86/609/CCE.

### Surgery

Experimental hemiparkinsonism was induced in rats by means of a unilateral lesion of the medial forebrain bundle, which was destroyed using a 6-hydroxydopamine (6-OHDA) injection. In brief, rats were anaesthetized with a ketamine/xylazine combination [75 mg/kg body weight ketamine hydrochloride/10 mg/kg body weight xylazine hydrochloride, intraperitoneally (i.p.); Sigma-Aldrich, St Louis, MO, USA] and immobilized in an adapted digital lab stereotaxic device (Stoelting Co., Wood Dale, IL, USA). Also, rats were treated with desipramine hydrochloride (10 mg/kg body weight; Sigma-Aldrich) 30 minutes before 6-OHDA injection to protect noradrenergic neurons from damage. An incision (0.5 cm) was performed in the skin of the skull to unilaterally lesion the right striatum with 6-OHDA (8 μg of 6-OHDA in 4 μl of saline solution containing 0.05% ascorbic acid; Sigma-Aldrich) by means of a Hamilton syringe (model 701, Reno, NV, USA). The stereotaxic coordinates, following the atlas of the rat brain (Paxinos and Watson, 2007), were, with respect to bregma: AP (anterior-posterior)=−2.2 mm, ML (medial-lateral)=−1.5 mm and DV (dorsal-ventral)=–7.8 mm (supplementary material Fig. S4). The 6-OHDA solution (or saline as a negative control; sham) was injected manually at a rate of 1 μl/min and, after the injection, the needle was left in place for 5 minutes before slowly retracting it to prevent reflux. Rats were then quickly warmed and returned to their cages. Finally, 3 weeks after the lesion, the extent of dopamine deafferentation was validated by assessing the rotating behavioural response to L-DOPA (3,4-dihydroxy-L-phenylalanine; Abcam Biochemicals, Cambridge, UK) administration. In brief, rats received an i.p. injection of L-DOPA (50 mg/kg body weight) in the presence of benserazide hydrochloride (25 mg/kg body weight, i.p.; Sigma-Aldrich) and the number of full contralateral turns counted during a 2-hour period. Dopamine deafferentation was considered successful in those animals that made at least 200 net contralateral rotations. Thereafter, animals were housed for 3 weeks before use.

### Fixed brain tissue preparation

Either rats or mice were anesthetized and perfused intracardially with 50–200 ml ice-cold 4% paraformaldehyde (PFA) in phosphate-buffered saline (PBS; 8.07 mM Na_2_HPO_4_, 1.47 mM KH_2_PO_4_, 137 mM NaCl, 0.27 mM KCl, pH 7.2). Brains were post-fixed overnight in the same solution of PFA at 4°C. Coronal sections (50–70 μm) were processed using a vibratome (Leica Lasertechnik GmbH, Heidelberg, Germany). Slices were collected in Walter’s Antifreezing solution (30% glycerol, 30% ethylene glycol in PBS, pH 7.2) and kept at −20°C until processing.

### Immunohistochemistry

Previously collected slices were washed three times in PBS, permeabilized with 0.3% Triton X-100 in PBS for 2 hours and rinsed back three times more with wash solution (0.05% Triton X-100 in PBS). The slices were then incubated with blocking solution [10% normal donkey serum (NDS) in wash solution; Jackson ImmunoResearch Laboratories, Inc., West Grove, PA, USA] for 2 hours at room temperature (RT) and subsequently incubated with the primary antibodies overnight at 4°C. After two rinses (10 minutes each) with 1% NDS in wash solution, sections were incubated for 2 hours at RT with the appropriate secondary antibodies conjugated with Alexa dyes (Invitrogen, Carlsbad, CA, USA), then washed (10 minutes each) two times with 1% NDS in wash solution and two more times with PBS, and mounted on slides. Fluorescence images of whole brain coronal sections were obtained using a SteREO Lumar.V12 fluorescence stereoscope (Carl Zeiss MicroImaging GmbH, Oberkochen Germany), whereas striatal images were captured using a Leica TCS 4D confocal scanning laser microscope (Leica Lasertechnik GmbH).

### Immunoelectron microscopy

Double-labelling post-embedding immunogold detection of A_2A_R and D_2_R was performed as previously described (Luján and Ciruela, 2001). Briefly, ultrathin sections 80-nm thick from Lowicryl-embedded blocks of striatum (see supplementary material Fig. S4) were picked up on coated nickel grids and incubated on drops of a blocking solution consisting of 2% human serum albumin (HSA) in 0.05 M TBS and 0.03% Triton X-100 (TBST). The grids were incubated with a mixture of goat anti-A_2A_R polyclonal antibody and rabbit anti-D_2_R polyclonal antibodies (10 μg/ml in TBST with 2% HSA) at 28°C overnight. The grids were incubated on drops of rabbit anti-goat IgG or goat anti-rabbit IgG conjugated to 10-nm and 15-nm colloidal gold particles, respectively (BBI Solutions, Cardiff, UK) in 2% HSA and 0.5% polyethylene glycol in TBST. The grids were then washed in TBS and counterstained for electron microscopy with saturated aqueous uranyl acetate followed by lead citrate. Ultrastructural analyses were performed in a Jeol-1010 electron microscope. Randomly selected areas were then photographed from the selected ultrathin sections at a final magnification of 50,000×. Then, the spatial distance between immunoparticles for A_2A_R and D_2_R was measured using appropriate software (ImageJ; NIH). To this end, we measured the nearest neighbour distances (NNDs) between the 10-nm gold particles (A_2A_R) and the 15-nm gold particles (D_2_R). Distances between the two particles were then compared between normal striatum and 6-OHDA-lesioned striatum using cumulative frequency plot and statistical analysis.

### Proximity ligation assay (PLA)

PLA, using the Duolink *in situ* PLA detection kit (Olink Bioscience, Uppsala, Sweden), was performed in a similar manner as immunohistochemistry explained above until the secondary antibody incubation step. The following steps were performed following the manufacturer’s protocol. Fluorescence images were acquired on a Leica TCS 4D confocal scanning laser microscope (Leica Lasertechnik GmbH) using a 60× N.A.=1.42 oil objective from the selected area (see supplementary material Fig. S4). High-resolution images were acquired as a *Z*-stack with a 0.2 μm *Z*-interval with a total thick of 5 μm. Nonspecific nuclear signal was eliminated from PLA images by subtracting DAPI labelling. The Analyze particle function from ImageJ (NIH) was used to count particles larger than 0.3 μm^2^ for PLA signal and larger than 100 μm^2^ to discriminate neuronal from glia nuclei. For each image the number of oligomer particles and neuron nuclei was obtained and the ratio between them was calculated. For all experiments, quantifications were performed from at least six images.

### Membrane preparations

To prepare rat and mice striatal membranes the procedure was the following: striatum either from normal or 6-OHDA-lesioned rats, or alternatively A_2A_R-KO mice (Ledent et al., 1997), was dissected and rapidly homogenized in ice-cold 10 mM Tris HCl (pH 7.4), 1 mM EDTA and 300 mM KCl buffer with Polytron at setting six for three periods of 10 seconds each. The homogenate was centrifuged for 10 minutes at 1000 ***g*** and the resulting supernatant centrifuged again for 30 minutes at 12,000 ***g***. The pellet was washed in Tris-EDTA buffer (10 mM Tris HCl, 1 mM EDTA, pH 7.4) and then resuspended in 15 ml of the same buffer containing 10% sucrose (wt/vol). It was then layered onto 15 ml of Tris-EDTA buffer with 35% sucrose (wt/vol). After centrifugation for 2 hours at 100,000 ***g***, membranes were collected at the 10–35% interface, and dispersed and washed in 50 mM Tris HCl (pH 7.4), 10 mM MgCl_2_.

### Time-resolved FRET assays

TR-FRET experiments were performed in polyornithine-coated, black-walled, dark-bottom, 96-well plates (Costar), on transiently transfected HEK-293 cells distributed at a density of 50,000 cells per well, on membrane preparations (20 μg per assay) from HEK-293, and on rat and mice striatal membrane preparations (70 μg per assay). To perform the taglite saturation assay between a SNAP substrate and a fluorescent ligand, adherent cells were washed and incubated with Tris-Krebs buffer (20 mM Tris-HCl, 118 mM NaCl, 5.6 mM glucose, 1.2 mM KH_2_PO_4_, 1.2 mM MgSO_4_, 4.7 mM KCl, 1.8 mM CaCl_2_, pH 7.4) containing 200 nM SNAP-Lumi4-Tb (GK) for 1 hour at 37°C. Cells were then washed three times with warm Tris-Krebs buffer. On the other hand, to detect TR-FRET between fluorescent ligands, cells or membranes were incubated with the fluorescent donor- and acceptor-labelled ligands in Tris-Krebs buffer. After an overnight incubation at 4°C, membrane preparations were washed by centrifugation at 12,000 ***g*** for 30 minutes, resuspended in the Tris-Krebs buffer and plated. Fluorescence and TR-FRET readings were performed using a PHERASTAR plate-reader (BMG Labtech, Durham, NC, USA). A 400-μs reading was measured after a 50-μs delay to remove the short-life fluorescence background from the signal. The specific TR-FRET signal was obtained at 665 nm for each assay, after subtracting the signals obtained for the negative control (incubation without donor or acceptor) and also the bleed-through from the donor into the 665 nm channel (incubation with only the donor).

### Statistics

The number of samples (*n*) in each experimental condition is indicated in figure legends. Statistical analysis was performed by Student’s *t*-test and by one-way ANOVA followed by Bonferroni’s multiple comparison post-hoc test. Statistical significance is indicated for each experiment.

## Supplementary Material

Supplementary Material
