# Supplementary material for: Untangling dopamine-adenosine receptor-receptor assembly in experimental parkinsonism in rats
Source: Dis Model Mech. 2014 Nov 14;8(1):57–63. doi: 10.1242/dmm.018143 (PMC4283650; doi:10.1242/dmm.018143)
Supplement: Supplementary Material [file supp_8_1_57__index.html]

Untangling dopamine-adenosine receptor-receptor assembly in experimental parkinsonism in rats — Supplementary Material 

# Untangling dopamine-adenosine receptor-receptor assembly in experimental parkinsonism in rats

## DMM018143 Supplementary Material

**Files in this Data Supplement:**

- **Supplementary Material**
