# Supplementary material for: Untangling dopamine-adenosine receptor-receptor assembly in experimental parkinsonism in rats
Source: Dis Model Mech. 2014 Nov 14;8(1):57–63. doi: 10.1242/dmm.018143 (PMC4283650; doi:10.1242/dmm.018143)
Supplement: Supplementary Material [file supp_8.1.57_DMM018143.pdf]

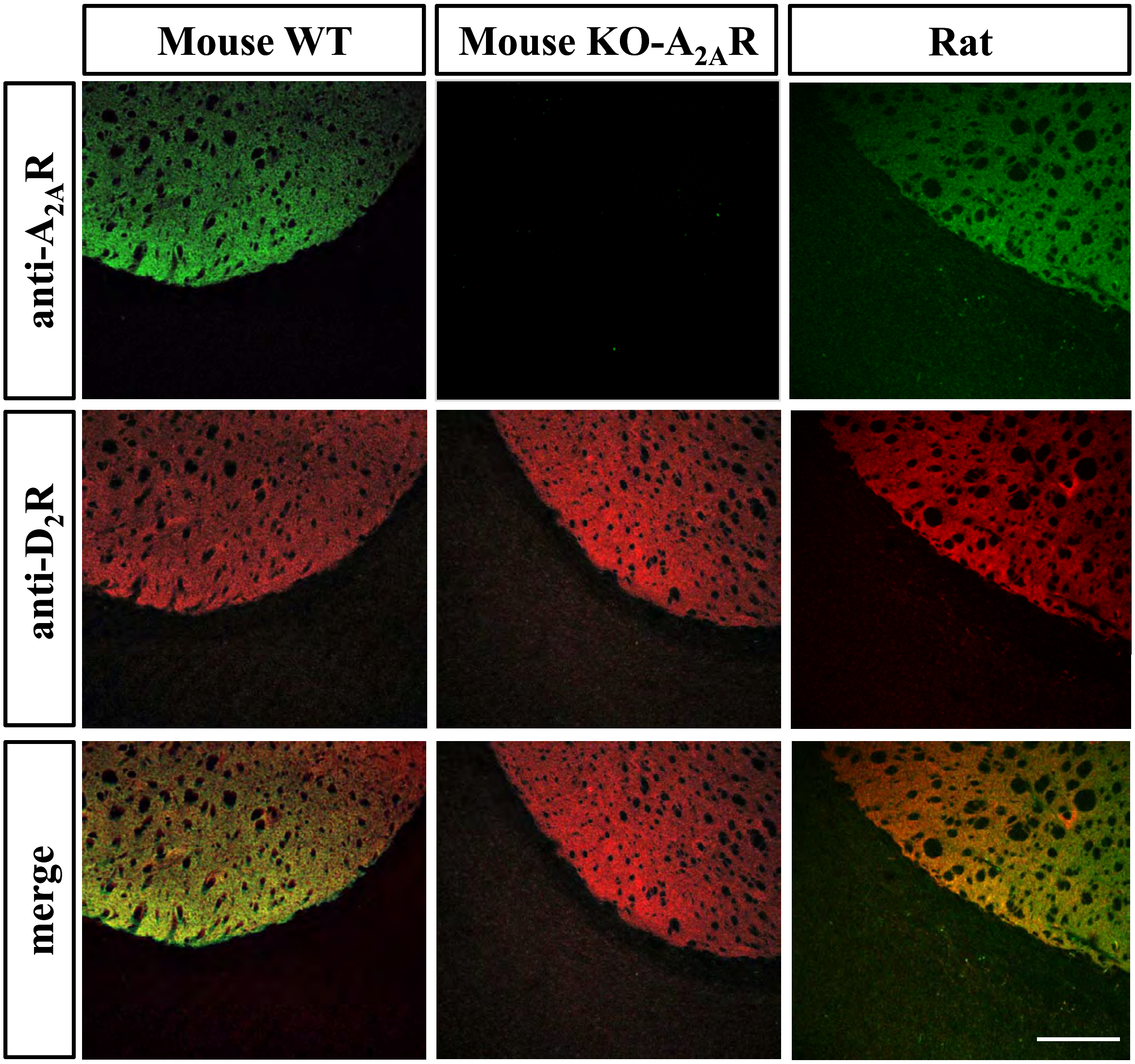

Supplementary Fig. S1. Specificity of the D_2R and $A_{2A}R$ antibodies. Representative images of D_2R and $A_{2A}R$ immunoreactivities in the dorsal striatum of wild-type (WT) (left) and $A_{2A}R$-KO mice (middle), and rats (right). Images revealed that both in WT mice and rats immunostaining was higher in the striatum compared with adjacent cortical areas. Conversely, $A_{2A}R$ immunostaining disappeared in $A_{2A}R$-KO mice (middle). Superimposition of images revealed a high receptor co-distribution in yellow (merge).

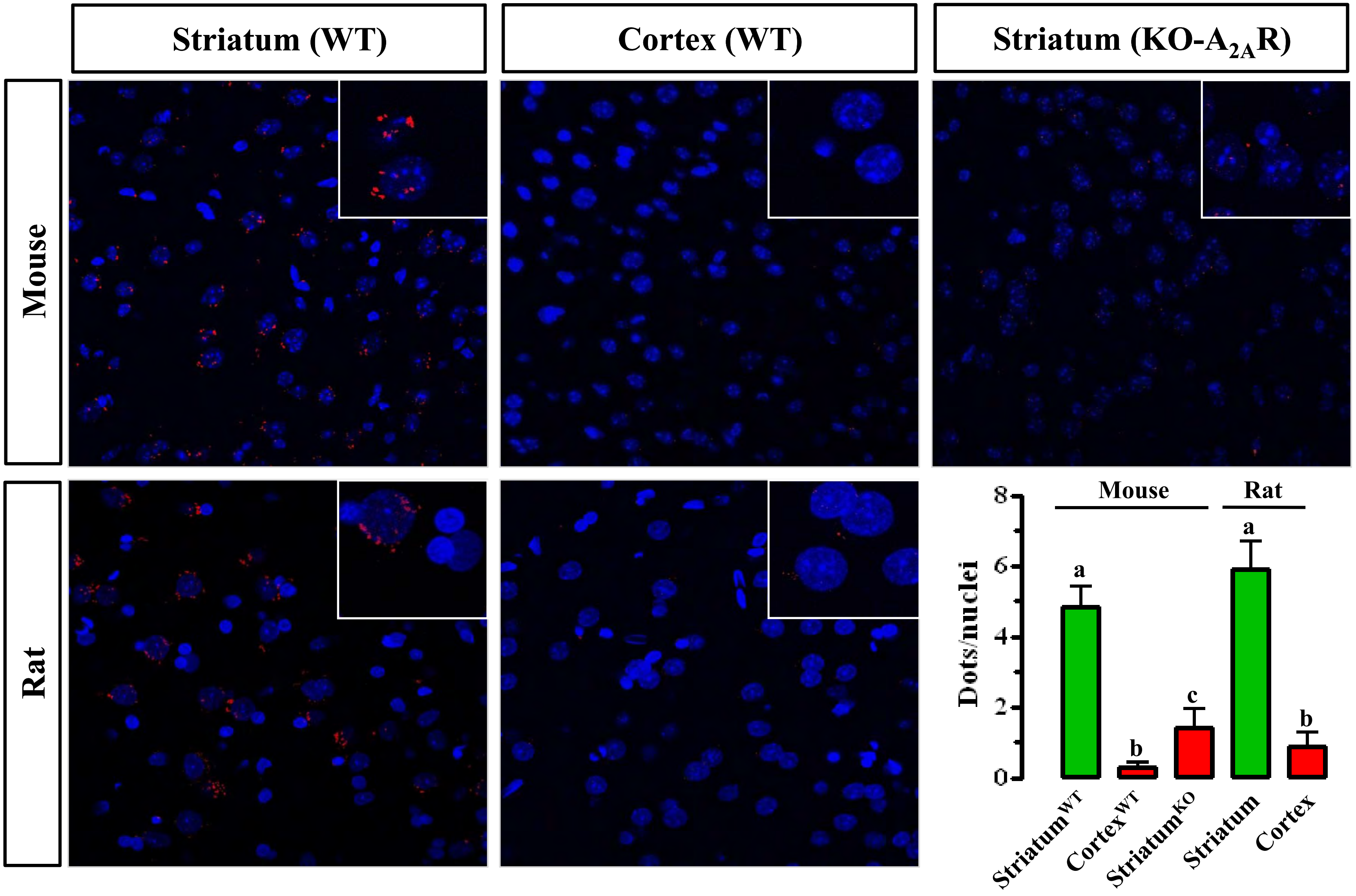

Supplementary Fig. S2. Specificity of the PLA signal. Representative images of dual recognition of D_2R and $A_{2A}R$ with proximity ligation assay, in striatum and cortex from wild-type (WT) (up) and rats (down). The PLA signal in striatums was significantly higher than in cortical areas ($P<0.05$). In addition, striatal sections of $A_{2A}R$-KO mice presented a significantly lower signal compared to that observed in WT mice ($P<0.05$). Values correspond to the mean ± s.e.m. (dots/nuclei) of at least 6 animals for condition.

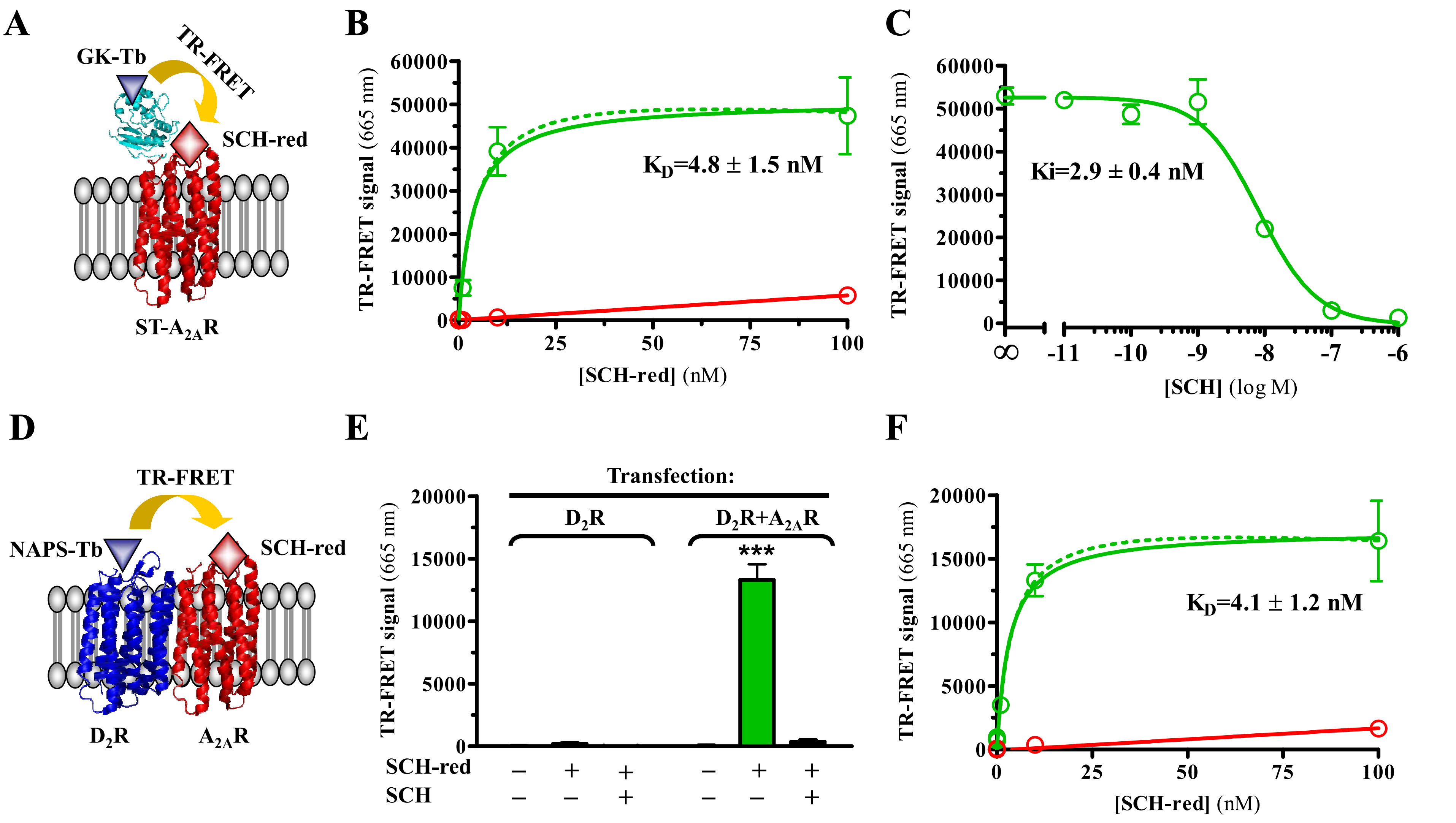

Supplementary Fig. S3. TR-FRET signals between fluorescent ligands bound to D_2R and $A_{2A}R$ expressed in heterologous systems. (a) Diagram illustrating the principle of TR-FRET between the ST-$A_{2A}R$ and the fluorescent antagonist SCH-red. (b) TR-FRET signal observed on HEK-293 cells transiently transfected with ST-$A_{2A}R$ receptors first labelled with the terbium labelled substrate (GK) and subsequently with increasing concentrations of the fluorescent $A_{2A}R$ antagonist SCH-red in the absence or presence of an excess of SCH (1 µM). The dotted green line represents the specific binding adjusted after subtracting to the total binding (green, in the absence of SCH) the unspecific binding (red, in the presence of SCH). (c) Inhibition of the TR-FRET signal by increasing concentrations of SCH (IC_{50}=8.10±0.2 nM). (d) Diagram illustrating the principle of TR-FRET between the fluorescent ligands bound to D_2R and $A_{2A}R$. (e) TR-FRET signal observed on HEK-293 cells transiently transfected either with h-D_2R alone or with h-D_2R and ST-$A_{2A}R$ receptors, and labelled with the fluorescent ligand NAPS-Lumi4-Tb (1 nM) alone, plus SCH-red (10 nM), or plus SCH-red (10 nM) and SCH (1 µM). (f) Variations in the TR-FRET signal as a function of acceptor concentration (SCH-red), in the absence or presence of an excess of SCH (1 µM). The donor ligand (NAPS-Lumi4-Tb) was maintained constant at 1 nM. The dotted green line represents the specific binding adjusted after subtracting to the total binding (green, in the absence of SCH) the unspecific binding (red, in the presence of SCH). Illustrated data are representative of at least three independent experiments performed in triplicate. Values correspond to the mean ± s.e.m. (AU, arbitrary units).

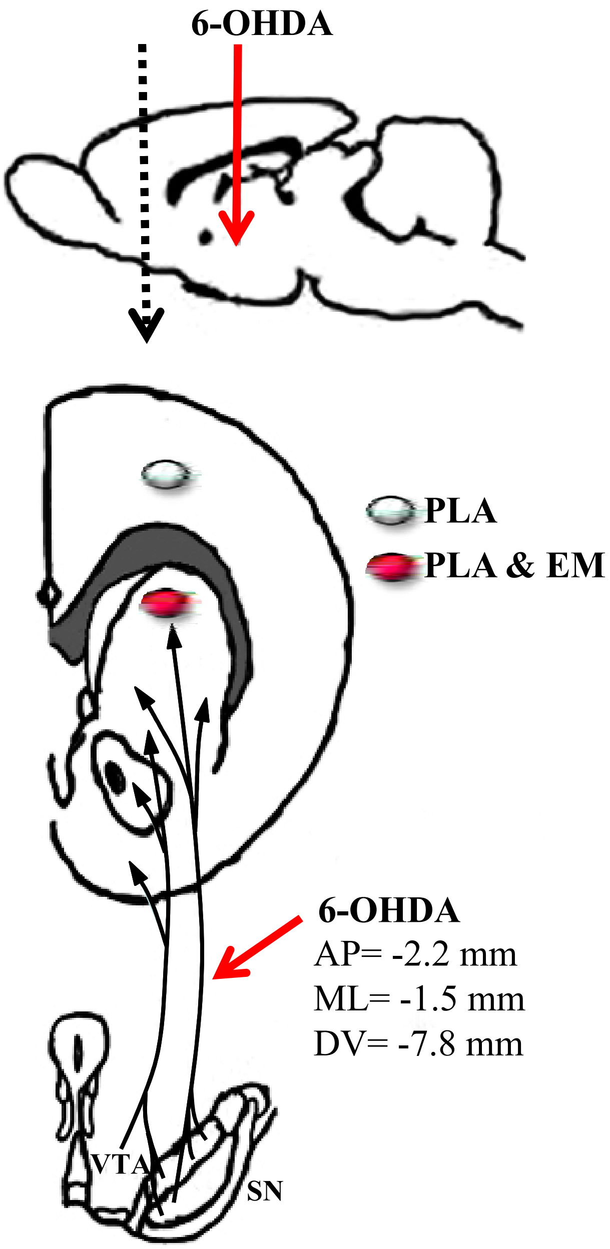

Supplementary Fig. S4. Schematic section of the rat brain (adapted from the stereotaxic atlas of Paxinos (Paxinos and Watson, 2007)). It is shown the placement of the 6-OHDA lesion into the left medial forebrain bundle (AP=-2.2 mm, ML=-1.5 mm and DV=-7.8 mm). The area where the proximity ligation assay (PLA) and immunogold-EM (EM) measurements were made is also indicated.
